# Second Opinions in Breast Cancer Surgery: What Have We Learned?

**DOI:** 10.7759/cureus.30180

**Published:** 2022-10-11

**Authors:** Meghan Beer, Hannah Allison, Carla Fisher, Betty Fan

**Affiliations:** 1 Breast Surgery, Community Health, Indianapolis, USA; 2 Center for Outcomes Research in Surgery, Indiana University School of Medicine, Indianapolis, USA; 3 Surgical Oncology, Indiana University School of Medicine, Indianapolis, USA

**Keywords:** treatment time, time-to-treatment, general surgery and breast cancer, breast cancer outcomes, second opinions

## Abstract

Introduction

Second-opinion consultations (SOCs) provide many benefits. However, duplicate office visits and the logistics of transferring medical records may be concerning for delaying treatment. There is currently no clear understanding regarding the characteristics of patients with breast cancer who desire second surgical opinions or if this contributes to delays in care.

Methods

A review of our institutional database from July 1, 2019, to December 31, 2019, identified breast cancer patients who were documented to be SOCs or primary consultations (PC). Neoadjuvant chemotherapy patients were excluded. Comparisons of patient characteristics, tumor characteristics, and surgery factors were performed using chi-square analysis. All analyses were two-tailed and statistical significance was assigned at p <0.05. This study was deemed IRB-exempt.

Results

In our review, 158 breast cancer patients were identified, 21 (13.3%) SOCs and 137 (86.7%) PCs. Of the SOCs, 90% (19/21) underwent surgery at our institution. The study revealed an increased incidence of SOCs in those patients who ultimately underwent mastectomy (p=0.039) as well as those with lower pathologic T stage (p=0.021). There were no other differences in demographics, surgery, or tumor characteristics. No delay was seen in time for treatment.

Conclusions

Patients who sought second opinions were more likely to undergo mastectomy and had lower pathologic tumor size. The time from biopsy to surgery appointment was longer in patients who sought second opinions but there were no differences in the time from biopsy or surgery appointment. It is encouraging that those who sought second opinions did not face any delay in care once established.

## Introduction

Second opinions in medicine are generally encouraged to assist patients with difficult medical decisions and reassure them of treatment plans [1]. The incidence of changes in diagnosis, treatment recommendations, and even prognosis from second opinions vary widely depending on specialty [2,3].

An estimated 284,200 individuals in the United States will be diagnosed with invasive breast cancer this year, and an additional 49,290 women will be diagnosed with non-invasive breast cancer [4]. Although this number has increased over the years, the risk of death due to breast cancer has decreased due to advances in earlier diagnosis and subsequent treatment regimens [4]. With constant evolution in treatment modalities, those diagnosed with breast cancer now have significantly more options in their care than in years past.

The myriad of decisions involved in the surgical treatment of breast cancer lead some patients to seek surgical second-opinion consultations (SOCs). Second opinions are often encouraged in breast cancer patients as an option to help patients better understand their treatment plans and feel confident in their decisions. They can also provide reassurance in confirming the diagnosis and proper treatment [5]. Despite the establishment of practice guidelines, the benefits of second opinions have not been replaced. Patients may want to verify that physicians are following said guidelines or simply want the reassurance of their decisions. Second opinions are also seen as a trend toward patient autonomy and taking control of their own treatment [6].

On the contrary, seeking a second opinion, particularly with a diagnosis of cancer, can be concerning as a cause for delaying treatment, given the possibility of duplicate office visits and the logistics of transferring medical records between institutions. This is particularly worrisome given studies in the past that have demonstrated that an increased preoperative time to surgery in patients with breast cancer is associated with a negative effect on overall and disease-specific survival [7].

There is currently no clear understanding regarding patients who desire second opinions. We sought to evaluate if there were any trends in patient demographics or tumor characteristics that may correlate with an increased likelihood to seek second surgical opinions. Furthermore, the authors of this study wanted to use this data to explore whether seeking a second opinion caused a delay in time to treatment.

## Materials and methods

Data collection

Our IRB-approved institutional Research Electronic Data Capture (REDCap) breast cancer database was used to identify new breast surgery clinic patients diagnosed with breast cancer from July 2019 to December 2019. These patients were seen by one of six surgeons at a large academic institution. Second-opinion patients include those who underwent surgery with us and those who chose to return to their initial institution. Breast cancer patients were classified as either SOC or primary consultations (PCs) based on chart review of surgeon consultation note. Second opinions were not specified in terms of the reason for the second opinion, i.e. patients included could have come for recommendations for surgery type, diagnosis, adjuvant treatment plans, etc. Those who received neoadjuvant chemotherapy were excluded.

Primary data were collected via chart review from our electronic medical record. Data variables collected included patient sociodemographics, medical history, tumor characteristics, and treatments performed. Treatments included the type of surgery and if reconstruction was performed. Receipt of adjuvant chemotherapy was also collected as well as genetic testing.

Relevant variables were stored electronically in the HIPAA-compliant REDCap application. REDCap is an electronic application developed by Vanderbilt University and is currently used by local end-users and collaborators from almost 5300 institutional partners to collect data for clinical research and to create data repositories for research projects. 

The quality control methods used included single entry with random checks of accuracy, and periodic extraction and data cleaning. To establish the reliability of the data collected in REDCap, we randomly selected 7% of the study sample and two persons (staff surgeon and data manager) entered the data for these subjects independently. Using Cohen’s kappa statistic for interrater reliability, we found the reliability measure to be high at around 0.965, hence satisfying the concordance and reliability of data entry. 

Data analysis

We used Stata statistical software version 16.1 for our analysis. For categorical variables, we performed chi-squared and Fisher's exact tests to examine bivariate relationships between patient characteristics and operative variables and the incidence of second-opinion visits. For continuous variables, we performed Kruskal-Wallis H tests to determine if there were any statistically significant differences between the SOC and PC groups. p-Values <0.05 were considered statistically significant. 

## Results

Patient and cancer characteristics

We identified 158 patients, 21 (13.3%) of those who were SOCs, and 137 (86.7%) who were PCs. Table 1 demonstrates the demographics of this group. There were no statistically significant differences in gender, race, education level, employment status, or insurance standing between those who sought SOC versus those who did not. Of the 21 patients in the SOC cohort, 17 patients (81.0%) lived outside the city of our institution.

**Table 1:** Patient demographics

Demographics	Second-opinion consultation	Primary consultation	Total	p-Value
	n=21 (13.3%)	n=137 (86.7%)	N=158 (100%)	
Gender				>0.999
Male	0 (0.0%)	0 (0.0%)	0 (0.0%)	
Female	21 (100.0%)	137 (100.0%)	158 (100.0%)	
Race				0.649
White	18 (85.7%)	117 (85.0%)	135 (85.4%)	
Black	2 (9.5%)	16 (11.7%)	18 (11.4%)	
Hispanic	0 (0.0%)	1 (0.7%)	1 (0.6%)	
Asian	1 (4.8%)	3 (2.2%)	4 (2.5%)	
Education level				0.677
Didn't complete high school	0 (0.0%)	1 (0.7%)	1 (0.6%)	
GED/High school diploma	4 (19.0%)	25 (18.2%)	29 (18.3%)	
College	3 (14.3%)	26 (19.0%)	29 (18.3%)	
Advanced degree	3 (14.3%)	9 (6.6%)	12 (7.6%)	
Unknown	11 (52.4%)	76 (55.5%)	87 (55.1%)	
Employment status				0.266
Full-time	8 (38.1%)	49 (35.8%)	57 (36.1%)	
Part-time	2 (9.5%)	4 (2.9%)	6 (3.8%)	
Disabled	1 (4.8%)	4 (2.9%)	5 (3.2%)	
Retired	5 (23.8%)	56 (40.9%)	61 (38.6%)	
Not employed	5 (23.8%)	16 (11.8%)	21 (13.3%)	
Self-employed	0 (0.0%)	5 (3.6%)	5 (3.2%)	
Unknown	0 (0.0%)	3 (2.2%)	3 (1.9%)	
City				0.212
Indianapolis	4 (19.0%)	48 (35.0%)	52 (32.9%)	
Other	17 (81.0%)	89 (65.0%)	106 (67.1%)	
Insured at diagnosis				0.133
Yes	20 (95.2%)	137 (100.0%)	157 (99.4%)	
No	1 (4.8%)	0 (0.0%)	1 (0.6%)	

Data regarding patient clinical and tumor characteristics are listed in Table 2. There were no differences in the age of patients between the SOCs and PC. Patients who sought SOC were more likely to undergo mastectomy compared to PCs (47.6% vs. 22.6%, p=0.039). Patients who sought SOC had lower pathologic tumor stages compared to the PC group, p=0.021. There was otherwise no significant difference in BMI, recurrence status, cancer status, clinical stage, genetics consultation, plastic surgery involvement, or chemotherapy usage (Table 2).

**Table 2:** Patient clinical data Age is given as mean (SD) and BMI as median (IQR). ALND, axillary lymph node dissection; SLNB, sentinel lymph node biopsy.

Clinical data	Second opinion consultation	Primary consultation	Total	p-Value
	n=21 (13.3%)	n=137 (86.7%)	N=158 (100%)	
Age at diagnosis	55 (48, 71)	61 (51, 71)	60.5 (48, 71)	0.380
BMI	29 (23.5, 34)	30 (25, 34)	30 (25, 34)	0.636
Recurrent cancer				0.753
Yes	4 (19.0%)	22 (16.1%)	26 (16.4%)	
No	17 (81.0%)	115 (83.9%)	132 (83.5%)	
Cancer status				0.141
Multifocal	5 (23.8%)	13 (9.5%)	18 (11.4%)	
Multicentric	1 (4.8%)	11 (8.0%)	12 (7.6%)	
Neither	15 (71.4%)	113 (82.5%)	128 (81.0%)	
Staging				
Clinical stage T				0.163
Tis	3 (14.3%)	32 (23.4%)	35 (22.2%)	
T1	12 (57.1%)	85 (62.0%)	97 (61.4%)	
T2	5 (23.8%)	10 (7.3%)	15 (9.5%)	
T3	0 (0.0%)	4 (2.9%)	4 (2.5%)	
T4	1 (4.8%)	2 (1.5%)	3 (1.9%)	
Unknown/Other	0 (0.0%)	4 (2.9%)	4 (2.5%)	
Initial stage N				0.999
N0	19 (90.5%)	119 (86.9%)	138 (87.3%)	
N1	1 (4.8%)	5 (3.6%)	6 (3.8%)	
N2	0 (0.0%)	1 (0.7%)	1 (0.6%)	
Unknown/Other	1 (4.8%)	12 (8.8%)	13 (8.2%)	
Pathologic stage T				0.021
Tis	1 (4.8%)	27 (19.7%)	28 (17.7%)	
T1	11 (52.4%)	85 (62.0%)	96 (60.8%)	
T2	5 (23.8%)	15 (10.9%)	20 (12.7%)	
T3	0 (0.0%)	3 (2.2%)	3 (1.9%)	
T4	0 (0.0%)	3 (2.2%)	3 (1.9%)	
Unknown/Other	4 (19.0%)	4 (2.9%)	8 (5.1%)	
Pathologic stage N				0.485
N0	12 (57.1%)	79 (57.7%)	91 (57.6%)	
N1	4 (19.0%)	11 (8.0%)	15 (9.5%)	
N2	0 (0.0%)	3 (2.2%)	3 (1.9%)	
N3	0 (0.0%)	1 (0.7%)	1 (0.6%)	
Unknown /Other	5 (23.8%)	43 (31.4%)	48 (30.4%)	
Type of procedure				0.039
ALND	0 (0.0%)	1 (0.7%)	1 (0.6%)	
SLNB	2 (9.5%)	4 (2.9%)	6 (3.8%)	
Lumpectomy	3 (14.2%)	35 (25.5%)	38 (24.1%)	
Lumpectomy and ALND	0 (0.0%)	1 (0.7%)	1 (0.6%)	
Lumpectomy and SLNB	4 (19.0%)	56 (40.9%)	60 (38.0%)	
Mastectomy, simple	7 (33.3%)	28 (20.4%)	35 (22.2%)	
Modified radical mastectomy	2 (9.5%)	2 (1.5%)	4 (2.5%)	
Mastectomy with reconstruction	1 (4.8%)	1 (0.7%)	2 (1.3%)	
Other	0 (0.0%)	3 (2.2%)	3 (1.9%)	
No procedure/surgery, consult only	2 (9.5%)	6 (4.4%)	8 (5.1%)	
Chemotherapy				0.999
Adjuvant	4 (19.0%)	27 (19.7%)	31 (19.6%)	
None	17 (81.0%)	110 (80.3%)	127 (80.4%)	
Plastics consultation				0.463
Yes	9 (43.9%)	46 (33.6%)	55 (34.8%)	
No	12 (57.1%)	91 (66.4%)	103 (65.2%)	
Reconstructive surgery				0.999
Yes	6 (28.6%)	40 (29.2%)	46 (29.1%)	
No	15 (71.4%)	97 (70.8%)	112 (70.9%)	
Genetic testing				0.244
Yes	11 (52.4%)	53 (38.7%)	64 (40.5%)	
No	10 (47.6%)	84 (61.3%)	94 (59.5%)	

Treatment delays

The time from initial biopsy to surgical consultation was significantly longer for the SOC group at 24 days (range: 13-29 days) compared to 13 days (range: 7-16 days) in those who were PC (p=0.002). Time from biopsy to surgery for SOC was 52 days (range: 27-81 days) compared to the PC group at 46 days (range: 33-55 days, p=0.924). Time elapsed between surgical consultation appointment to surgery in the SOC group was 31 days (range: 19-37 days) compared to those in the PC group at 33 (range: 20-41 days, p=0.280) (Table 3).

**Table 3:** Time to treatment and appointments The values provided here are represented as median (IQR).

Outcomes	Second opinion consultations	Primary consultations	Total	p-Value
	n=21 (13.3%)	n=137 (86.7%)	N=158 (100%)	
Time from biopsy to surgery appointment	24 (13, 29)	13 (7, 16)	15 (7, 17)	0.002
Time from biopsy to surgery	52 (27, 81)	46 (33, 55)	46 (33, 56)	0.924
Time from surgery appointment to surgery	31 (19, 37)	33 (20, 41)	33 (20, 40)	0.280

## Discussion

Second opinions can impact breast cancer care and treatment. Reported rates of second opinions in oncology ranged from 1% to 88% [8]. Several recent studies have examined the effect of discrepancies in care plans among oncology specialties. Heeg et al. found that nearly 60% of discrepancies in diagnostic and treatment proposals were categorized as major: neoadjuvant systemic treatment instead of primary surgery, breast-conserving surgery instead of mastectomy, and proposing postmastectomy immediate breast reconstruction [9]. Moreover, several studies have demonstrated that second opinions in pathology demonstrated clinically significant discrepancies, which could drastically affect treatment decisions [10-12]. This data further illustrates the variability among practitioners in breast cancer care and the impact this has on outcomes. Second opinions can, therefore, be invaluable to patients as a tool for validating their treatment plans and seeking out their individual options. A recent second-opinion program for breast cancer found that management plans were different in 20.3% of patients and that most commonly the difference was eligibility for breast conservation in patients who were offered only mastectomy [13].

Overall, in our study, there were no differences in age between the SOC and PC. This is different than many studies which indicate younger patients are more likely to seek second opinions. A questionnaire-based study of patients seeing a medical oncologist at the Sydney Cancer Centre between January 2006 and January 2008 found that patients seeking a second opinion were typically more educated, younger, and female. The authors postulated that this was likely due to preferences for more detailed information [14]. Similarly, among second opinions at a colorectal cancer clinic, those who were second-opinion patients tended to be younger compared to those coming for PC [15]. Additionally, there was no difference identified in study cohorts about the level of education. This is contrary to other known studies. A meta-analysis of 25 studies regarding second opinions in oncology found that higher education was most consistently seen in those who sought second opinions [8]. Our results were likely influenced by a smaller sample size, and a greater difference may be seen in a larger study.

Of those who sought SOCs, the majority had a lower pathologic tumor stage compared to PC patients, p=0.021. The authors suspect that one explanation is due to those patients with earlier-stage tumors having more time to explore surgical and treatment options (i.e. lumpectomy vs. mastectomy), compared to those with later-stage tumors where a more algorithmic approach is often taken and patient choices are more limited. This difference in tumor size stage was not seen in our clinical-stage analysis. The authors suspect this was due to sample size limitations.

In this study, patients who sought second opinions were more likely to have a mastectomy compared to those who were seen in PC (47.6% vs. 22.6%). This is consistent with several studies, which have historically demonstrated that women more often seek surgical second opinions when a mastectomy is recommended [16,17]. It is possible that patients sought second opinions when seeking a more complex oncoplastic procedure and/or reconstructive options but this was not reflected in our study results. This difference may be further examined in a larger sample size. Conversely, it may be simply the need for a mastectomy that is the sole driver of patients seeking an SOC and additional confirmation before committing to more extensive and irreversible surgery.

Most importantly, despite a statistically significant increase in the timing of initial biopsy to surgical appointment seen in the SOC group, there was no increase in time from biopsy to surgery nor time from surgical consultation to surgery. Surprisingly, patients who were seen as SOC had a shorter time from surgical consultation to surgery compared to those seen as PC. We suspect that given the proactive awareness of second opinions already experiencing a delay from biopsy and diagnosis to the surgical appointment, increased efforts were made to help minimize the delays further once care was established. Another theory is that since part of the SOC patients’ workup is often at least partially started at another institution, tests such as MRIs or genetic testing, which historically can contribute to delays, may already have been performed or scheduled which would help with expeditious scheduling. Meaningfully, physicians should continue to be supportive of patients who desire to seek second opinions as fear of treatment delays may not be as worrisome as initially assumed.

Limitations

There was intrinsic selection bias given that the study only examined patients seen as second opinions at our institution and did not include those patients who sought second opinions at outside facilities. Additionally, this study focused on the population of patients inherent to our geographic location. It is unclear whether these results can be extrapolated to a more general population. Our statistical analysis was also limited by the small cohort size.

## Conclusions

In our study, patients who sought second opinions were more likely to undergo mastectomy and have a smaller pathologic tumor stage. There were no other clear trends in our data. However, more importantly, our study found that SOC did not delay the time to treatment. While more granular data are needed to further explore this patient subset, it is reassuring that those who sought second opinions did not face any delay in care once established.
